# Absenteeism Among Healthcare Workers: Job Grade and Other Factors That Matter in Sickness Absence

**DOI:** 10.3390/ijerph22010127

**Published:** 2025-01-20

**Authors:** Carine J. Sakr, Lina M. Fakih, Umayya M. Musharrafieh, Ghassan M. Khairallah, Maha H. Makki, Rita M. Doudakian, Hani Tamim, Carrie A. Redlich, Martin D. Slade, Diana V. Rahme

**Affiliations:** 1 Employee Health Unit, Department of Family Medicine, Faculty of Medicine, American University of Beirut Medical Center, Beirut P.O. Box 11-0236, Lebanon; cs56@aub.edu.lb (C.J.S.); lf32@aub.edu.lb (L.M.F.); um00@aub.edu.lb (U.M.M.); gk44@aub.edu.lb (G.M.K.); rd01@aub.edu.lb (R.M.D.); 2 Clinical Research Institute, Faculty of Medicine, American University of Beirut, Beirut P.O. Box 11-0236, Lebanon; mm209@aub.edu.lb (M.H.M.); htamim@aub.edu.lb (H.T.); 3 Yale Occupational and Environmental Medicine Program, Yale University School of Medicine, Yale-New Haven Hospital, Yale University, New Haven, CT 06501, USA; carrie.redlich@yale.edu (C.A.R.); martin.slade@yale.edu (M.D.S.)

**Keywords:** healthcare workers, sickness absenteeism, sick leave predictors, job grade, tailored interventions, health inequity

## Abstract

Background: Absenteeism among healthcare workers (HCWs) disrupts workflows and hampers the delivery of adequate patient care. The aim of the study was to examine predictors of sick leaves among HCWs in a tertiary medical center in Lebanon. Methods: A retrospective analysis of sick leaves linked to health records of 2850 HCWs between 2015 and 2018 was performed. Sick leave episodes were stratified by diagnosis. Bivariate and negative binomial regression analyses were performed to investigate predictors. Results: The mean number of sick leave episodes was 10.6 per person over 4 years. The strongest predictor of higher sickness absenteeism was low job grade (IR = 1.52; 95% CI = 1.39, 1.67). Female sex (IR = 1.24; 95% CI = 1.14, 1.36), older age (IR = 1.19; 95% CI = 1.08, 1.30), being married (IR = 1.21; 95% CI = 1.11, 1.33), being a current smoker (IR = 1.21; 95% CI = 1.11, 1.32), and having a history of selected medical conditions were all significant sick leave predictors. Conclusion: Demographic, work-related, and health-related predictors are associated with the number of sick leave episodes. To address the health inequity, additional research should evaluate how some socio-economic factors determine poorer health outcomes and should guide approaches to address this crucial issue to protect the health and well-being of this key workforce.

## 1. Introduction

Sick leave is a significant public health problem and can be used as a measure of ill health [1]. It can serve as an indicator of the health of individuals of working age, as it provides insights into their overall productivity and well-being [2]. Healthcare workers (HCWs) are essential for the delivery of care for patients, and sick days can greatly impact staffing, the quality of care, and work efficiency [3]. To implement interventions to reduce excessive sick leave absences, it is important to identify their predicting factors. Several studies have looked at the predictors of sick leave episodes in hospital settings, primarily among nurses. Age, education, job type, work shift, time spent in the institution, and workplace conditions have been found to be associated with sick leave among nurses [4]. In recent years, infectious diseases such as the COVID-19 pandemic have resulted in an increase in sick leave episodes among HCWs [5,6]. In a study conducted in Lebanon, age and job position were predictors of longer COVID-19-related sick leaves [7].

Most studies on sick leave absences have been performed in high-income countries and have focused on nurses [8]. Data available on sick leave episodes from middle- and low-income countries is often incomplete or inaccurate [8]. Healthcare delivery requires a diverse workforce beyond nurses, including radiology and laboratory technicians, pharmacists, nursing assistants, clerks, dietary workers, transport service workers, and housekeepers, among many others.

The aim of this study was to examine the multiple predictors of sick leave over a 4-year period among a large and diverse sample of HCWs who had sickness-related absenteeism. We hypothesized that sick leave episodes would be associated with multiple demographic, socio-economic, occupational, and health-related factors.

## 2. Materials and Methods

### 2.1. Study Design, Setting, and Data Sources

This was a retrospective cross-sectional analysis study in which we reviewed the sick leave records of employees of the American University of Beirut Medical Center over a four-year period ranging from 2015 to 2018. Medical information, including diagnosis for the sick leave, duration of sick leave, past medical history, smoking, alcohol consumption, exercise, and blood pressure (systolic and diastolic), were retrieved from the university health system (UHS) electronic health record (as recorded by clinicians during medical visits). Demographic information, including date of birth, sex, job position, department, marital status, and grade, were provided by the Human Resources Department. Analyses were performed on de-identified datasets. The research protocol was approved by the American University of Beirut Institutional Review Board (IRB) (protocol number SBS 2018-0236). Waiver of informed consent was granted by the IRB, as the study was a secondary data analysis of anonymous records and posed minimal risk to participants. The research practices were compliant with the Helsinki Declaration.

### 2.2. Data Collection and Inclusion and Exclusion Criteria

We reviewed all sick leave records of employees working at American University of Beirut Medical Center. The university policy on sick leave specifies that all employees and workers are eligible for sick leave. Sick leaves are granted to all employees and workers in cases of illness, injury, pregnancy, temporary disability, dental or medical treatment, and exposure to contagious diseases requiring isolation of employees.

All HCWs who took at least one sick leave episode between January 2015 and December 2018 were included in the study. These included medical personnel, nurses, administrative, housekeeping, food service, laboratory, medical engineering, plant engineering, respiratory therapy, physical therapy, endoscopy, outpatient labs, and security staff. The total number of employees included in our study was 2850, which represents two-thirds of the total workforce. A sick leave episode was defined as each occurrence where an employee was absent from work for at least one day. The number of sick leave times was defined as the cumulative number of sick leave episodes taken throughout the whole study period. Physicians were excluded from our study sample since it has been well documented in previous studies that this occupational group tends not to take sick leave [9,10].

### 2.3. Variables

Demographic variables such as sex, age, marital status, and grade were coded as binary: sex (male/female), age (less than 35 years old/35 years and above), marital status (married/not married), and grade (lower-grade (G) positions G1–G8 for employees with a lower education level, lower salary, and non-managerial positions; managerial positions G9–G13). Job position was grouped by the research team into 12 categories whereby people performing similar jobs were classified within the same category.

As for medical history variables, past medical conditions were coded as binary yes/no variables. A similar coding was performed for lifestyle factors such as alcohol and exercise (any level of exercise and alcohol intake were coded as “yes”). Smoking status was categorized as current smoker, ex-smoker, and non-smoker (depending on what the patient reported to their clinician during the medical encounter). The data were recorded and kept updated continuously with each medical visit by the clinician. The research team classified sick leave diagnoses into 13 categories, and similar diagnoses groups were placed under the same category.

### 2.4. Statistical Analysis

Data were entered into the Statistical Package for Social Sciences (SPSS, version 22), which was used for data cleaning, management, and analyses. Descriptive statistics were summarized by presenting the number and percentage for categorical variables and mean and standard deviation for continuous variables. To identify the predictors of the number of sick leave episodes, a negative binomial regression analysis was run, including variables with a *p*-value of <0.2 at the bivariate analysis level.

The model included variables that are known to be associated with sick leave, such as sex. Correlations were calculated between each set of independent variables using Spearman’s correlation. If the magnitude of the correlation was greater than 0.4, only one of the variables was included in the analysis, as this was interpreted as a moderate correlation between variables based on Dancey and Reidy [11]. The correlation between job position and grade was evaluated. A *p*-value of <0.05 was considered statistically significant in the final model.

## 3. Results

Table 1 shows the descriptive statistics of the study population (individuals with at least one sick leave episode throughout the study period, *n* = 2850). The mean number of sick leave episodes was 10.6 occurrences per person over a 4-year period. A total of 52.6% of participants were female, and around half the study population was aged 35 years or older. The most common type of HCW was registered nurses (RNs) (30.6%), followed by clerks and clinical assistants (CAs) (26.5%) and orderlies, practical nurses (PAs), and nursing assistants (NAs) (15.9%). The most common medical conditions among the participants were respiratory problems (62.3%) and gastrointestinal problems (42.8%). In total, 49.1% of the study population reported that they currently smoked.

When looking at the distribution of sick leave diagnoses, infectious diseases and musculoskeletal disorders were responsible for the highest number of sick leave episodes: 6598 (21.7%) and 6097 (20.0%), respectively (Table 2). The mean duration of sick leave ranged from 2.4 days (genitourinary, gynecological and obstetrics diagnosis) to 2.9 days (respiratory illness diagnosis). All diagnoses had a short sick leave duration on average.

Bivariate analysis was run between the number of sick leave times and each predictor. Predictors with a *p*-value of <0.2 were chosen to be entered in the negative binomial regression model, and these included age, grade, marital status, certain medical history conditions (gastrointestinal problems, back pain, chest pain, headache, respiratory problems, diabetes, and mental health problems) in addition to lifestyle factors such as smoking and exercise (Table 3). Sex was also included due to its importance.

Grade and job position were highly correlated (Pearson’s chi-square = 1349.76, *p*-value < 0.001). Each of these two variables was run separately in the multivariate model. Both models showed goodness of fit. The model with grade had the lowest Akaike’s Information Criterion (AIC) and was therefore selected as the final model. In the final multivariate regression analysis, job grade was the strongest determinant of higher sick leave episodes. The incidence rate of sick leave episodes among HCWs of lower grades was 1.59 times higher than those of higher grades (IR = 1.52; 95% CI = 1.39, 1.67). In addition, the following variables remained significant predictors of taking a higher number of sick leave episodes: female sex (IR = 1.24; 95% CI = 1.14, 1.36), older age (IR = 1.19; 95% CI = 1.08, 1.30), and being married (IR = 1.21; 95% CI = 1.11, 1.33). As for medical history, the following medical conditions remained significant in the adjusted model: back pain, headache, respiratory problems and allergies, gastrointestinal problems, and mental health problems. Finally, being a current smoker (IR = 1.21; 95% CI = 1.11, 1.32) was also a significant predictor (Table 4).

## 4. Discussion

In this study that aimed to examine determinants of HCW sick leave, many demographic, socio-economic, and medical factors were found to be significant predictors of sickness absenteeism among HCWs, the strongest being job grade. Female sex, older age, being married, and being a current smoker were significant predictors of more sick leave episodes. In addition, working in lower-grade jobs and having a history of certain medical conditions (particularly gastrointestinal problems, mental health problems, back pain, headache, and respiratory problems) also predicted a higher number of sick leave episodes.

Our results compare to previous literature on predictors of sick leave among HCWs, which confirm that the female sex, older age, marital status, and working in lower-grade positions predict more sick leave episodes. Studies including all hospital staff report that women are more absent from work than men [12]. A higher prevalence of sick leave is also seen among older age workers [13]. A similar trend was observed among married workers [3]. Moreover, the level of hierarchy has been reported as a predictor of sick leave among HCWs. It has been found that HCWs who are higher in the hierarchy, such as doctors, report less sick leave compared with other workers [8]. In Finland, doctors had lower rates of sick leave compared with nurses [14]; yet, in other studies, auxiliary staff showed the highest sick leave rates and duration compared with medical and dental staff [15]. These findings were confirmed by studies from other countries [8].

Our findings echo the previous body of evidence suggesting that the lowest-paid workers have poorer health outcomes and more sick leaves than other highest-paid workers. A cohort study that followed up hospital employees over a year showed that sickness absences increased with lower socio-economic status [16]; however, this relationship is still not well understood since many factors and possible mediators can interplay. Examining these factors can help in understanding how low socio-economic status is associated with more sickness absenteeism at work. Low job and worktime control [17], high job strain [18], and physically demanding jobs [19] can possibly influence or explain this relationship. According to the results of one systematic review, job dissatisfaction and psychosocial stressors can also increase sick leaves due to mental health disorders [20].

In addition, low-paid jobs that are more physically demanding might entail more exposure to safety hazards; therefore, workers are more prone to acute physical injuries and, hence, sick leaves. A study showed that a lower socio-economic status is associated with more work injury absences [21]. The inequalities in work injury absence were larger than in all-cause sickness absence [21].

Since most of the mentioned factors are modifiable, interventions can be developed at the workplace to mitigate their effect [22]. However, other non-modifiable factors were identified in the literature, adding to the complexity of the issue. A twin study found that despite low education and income being associated with more sick leaves at work, this relationship is attenuated in dizygotic twin pairs and non-significant in monozygotic twin pairs [23]. Hence, the observed association might be confounded by other factors, one of which could be genetic [23].

In addition to socio-economic and occupational factors, our study also examined the past medical history of HCWs and lifestyle factors such as smoking and exercise. HCWs with a history of gastrointestinal problems, mental health problems (particularly anxiety and depression), back pain, headache, or respiratory problems were found to have a higher number of sick leave episodes compared with those who did not have these conditions. These findings are consistent with previous studies that examined the association between chronic health conditions and sick leave among workers. Prior to the COVID-19 pandemic in Qatar, HCWs with respiratory-related problems, back and/or neck-related problems, and gastroenteritis problems took a higher number of sick leaves [2]. In other settings, the main conditions associated with the frequency and duration of sick leave episodes among HCWs were musculoskeletal disorders, flu, infectious diseases, and mental and behavioral disorders [3,24]. Studies from different parts of the world showed an increasing trend in the number of sick leave days with an increase in the number of chronic conditions [25]. Moreover, mental health disorders were found to be strong predictors of sickness absence [26], with anxiety being the second most common type of mental health disorder in some settings, with a prevalence of 15.9% [26].

Our results show that sick leave numbers were also higher among HCWs who currently smoke compared with those who do not or those who used to, even after adjusting for age, work-related factors, and health conditions. This finding is concordant with previously published literature. A systematic review of the association between smoking and sickness absence found that smoking was associated with both a higher risk and a higher number of sick leave days [27]. The review, however, only included four studies from the healthcare sector and did not have sufficient data on the duration of sickness absences [27].

### 4.1. Strengths and Limitations

One limitation of our study is the restriction to one medical center. Our sample is likely comparable to other major medical centers in developed countries but may not be comparable to smaller hospitals in different settings. In fact, evidence has shown that occupational health and safety standards and accreditation status differ significantly in Lebanon between hospitals of different sizes and types (private versus public) [28]. In addition, other occupational and demographic variables such as work shift, age at recruitment, years of employment, and having children were missing from our database. Data on the work-relatedness of sick leave episodes was also not available and, therefore, not analyzed. However, it can be inferred from our data on the distribution of sick leaves by diagnoses that the overwhelming majority is non-work-related. Moreover, lifestyle factors (exercise and alcohol use) were only coded as dichotomous variables, which limited our ability to investigate more deeply the association between sick leaves and the level of physical activity or the amount of alcohol consumption. In addition, our study mainly focused on individuals who had at least one sick leave episode. This approach does not identify predictors of sick leave occurrence in general but rather predictors of more frequent sick leave episodes among those who already took them. Finally, our data show that both the number of sick leave episodes and their duration were low, specifically for mental health problems. This can be a potential under-reporting of mental illnesses due to fear of stigma in our Middle Eastern cultural context [29].

Despite those limitations, this study had many strengths worth emphasizing. First, it involved a large sample size of HCWs who took sick leaves (*n* = 2850) over a 4-year period. Second, while other studies focused on specific health professions such as nurses or physicians only, our study involved a comprehensive sample of the totality of the hospital workforce from diverse social gradients. Our sample of HCWs included physicians, registered nurses, nursing assistant personnel, administrative, housekeeping, food service, laboratory, medical engineering, plant engineering, respiratory therapy, physical therapy, endoscopy, outpatient labs, and security staff. Third, our large database included all sick leave records (work-related and non-work-related), as well as medical records. This allowed us to analyze past medical history as a potential predictor of sick leave. It is also important as it identified other modifiable risk factors, which is important in designing interventions to reduce sick leave among HCWs. Fourth, unlike other studies that mainly focused on selected predictors analyzed separately, the strength of this study lies in its multivariate analysis that allowed for the simultaneous inclusion and assessment of multiple relevant predictors (socio-economic, demographic, occupational, lifestyle, physical and mental health conditions) of sickness absenteeism. Our findings highlight the importance of addressing socio-economic inequities when tailoring sick leave interventions, among other factors.

### 4.2. Recommendations

The findings of our study can inform future interventions and practice. Modifiable risk factors, especially occupational ones (job dissatisfaction, job strain, low job and worktime control), in addition to lifestyle factors (smoking), should be considered to attenuate the effect of low job grades on sickness absenteeism. To address health inequity, additional research should evaluate how some socio-economic factors determine poorer health outcomes. More research efforts should focus on mediation analysis to better understand how the different factors interplay, especially in resource-constrained settings.

## 5. Conclusions

Our study found a positive association between several risk factors (socio-economic, demographic, and health-related) and the number of sick leave episodes, the strongest being low job grade. It is crucial to understand these risk factors to address the reasons underlying sickness absenteeism among HCWs. Specific interventions are needed to guide policy and practice for healthier and more productive workplaces. The global shortage in HCWs [30] calls for action to address sickness-related absence. The results of this study underscore the need to tailor sick leave interventions to different groups of workers, especially among those at the lowest socio-economic gradient, to avoid health inequities.

## Figures and Tables

**Table 1:** Descriptive statistics of healthcare workers with sick leave episodes (*n* = 2850).

Variable	Mean ± Standard Deviation
**Sick leave episodes**	10.6 episodes ± 10.6
**Sick leave duration**	2.60 days ± 4.5
**Variable**	**Frequency (%)**
**Sex**	
Female	1499 (52.6%)
Male	1351 (47.4%)
**Age**	
Less than 35 years old	1476 (51.8)
35 years and older	1374 (48.2)
**Marital Status**	
Married	1686 (59.2%)
Not married	1164 (40.8%)
**Grade**	
G9 to G13+	1541 (54.1%)
G1 to G8	1309 (45.9%)
**Position**	
RN	871 (30.6%)
Clerk—CA	755 (26.5%)
Orderly—PN-NA	454 (15.9%)
Physical plant, laundry, Central Sterilization Department, motor pool (parking services)	205 (7.2%)
Laboratory technician	164 (5.8%)
Housekeeping	124 (4.4%)
Dietary	75 (2.6%)
Other (physical therapist, radiology, respiratory therapist, pharmacist, other)	202 (7.1%)
**Medical History**	
Respiratory problems	1787 (62.7%)
Gastrointestinal problems	1198 (42.0%)
Back pain	816 (28.6%)
Headache	293 (10.3%)
Dizziness	230 (8.1%)
Anemia	220 (7.7%)
Mental health (depression and anxiety)	153 (5.4%)
Hypertension and kidney disease	130 (4.6%)
Chest pain	74 (2.6%)
Diabetes	34 (1.2%)
**Smoking status**	
Yes—current smoker	1378 (49.1%)
Yes—ex-smoker	67 (2.4%)
No	1363 (48.5%)
**Exercise**	
Yes	1351 (55.2%)
**Alcohol**	
Yes	492 (17.3%)

G: grade; CA: clinical assistant; PN: practical nurse; RN: registered nurse.

**Table 2:** Distribution of sick leave episodes and duration of sick leave by diagnosis.

Sick Leave Diagnosis	Number of Episodes * *n* (%)	Sick Leave Duration (in Days) Mean ± Standard Deviation
Infectious diseases (upper respiratory infections, acute gastroenteritis, cellulitis)	6598 (21.7%)	2.6 ± 5.0
Musculoskeletal disorders	6097 (20.0%)	2.6 ± 5.1
Surgeries/injuries	4792 (15.7%)	2.5 ± 4.5
Non-infectious gastrointestinal problems	3714 (12.2%)	2.6 ± 4.5
Other diagnoses ^&^	3175 (10.4%)	2.5 ± 4.4
Genitourinary, gynecology obstetrics	1612 (5.3%)	2.4 ± 3.9
Neurological, psychological, and behavioral problems	1270 (4.2%)	2.6 ± 6.2
Dental problems	931 (3.0%)	2.7 ± 5.1
Non-infectious respiratory illness	629 (2.0%)	2.9 ± 6.6
Ophthalmology	624 (2.0%)	2.8 ± 4.6
Cardiovascular diseases	395 (1.3%)	2.3 ± 3.2
Ear, nose, and throat conditions (ENT)	331 (1.0%)	2.6 ± 4.8
Dermatology	250 (0.8%)	2.3 ± 3.5
Total	30,418	

* The number of sick leave episodes taken in the 4-year period, ^&^ other diagnoses include metabolic, congenital, hematology/oncology, health maintenance, general symptoms, and non-specified diagnoses.

**Table 3:** Bivariate analysis of the number of sick leave episodes with all potential predictors.

		Number of Sick Leave Episodes Mean ± Standard Deviation	*p*-Value
**Sex**	Male	10.3 ± 10.6	0.268
	Female	10.8 ± 10.6	
**Age**	Less than 35 years old	9.1 ± 9.2	<0.001 *
	35 years or older	12.1 ± 11.8	
**Marital status**	Married	11.6 ± 11.1	<0.001 *
	Not married	9.1 ± 9.7	
**Grade**	G1 to G8	13.5 ± 12.3	<0.001 ***
	G9 to G13	8.1 ± 8.2	
**Position**	Clerk-CA	8.5 ± 9.0	<0.001 *
	RN	10.3 ± 9.5	
	Orderly-PN-NA	13.8 ± 12.3	
	Housekeeping	14.3 ± 14.5	
	Dietary	14.4 ± 14.2	
	Lab Technician	8.2 ± 8.6	
	Physical plant, laundry, CSD, motor pool	14.2 ± 12.9	
	Physical therapist	6.9 ± 5.5	
	Radiology technician	6.9 ± 7.4	
	Respiratory therapist	9.3 ± 6.4	
	Pharmacist	6.1 ± 5.9	
	Other	5.3 ± 6.5	
**Medical History**			
**Respiratory problems (cough, bronchitis, allergy, other respiratory problems)**	Yes	11.45 ± 10.951	<0.001 ***
	No	9.05 ± 9.884	
**Gastrointestinal problems (diarrhea, irritable bowel syndrome, abdominal pain)**	Yes	11.88 ± 11.19	<0.001 ***
	No	9.59 ± 10.09	
**Back pain**	Yes	13.28 ± 11.45	<0.001 ***
	No	9.46 ± 10.07	
**Headache**	Yes	12.2 ± 10.8	0.006 ***
	No	10.4 ± 10.6	
**Dizziness**	Yes	11.1 ± 9.7	0.409
	No	10.5 ± 10.7	
**Anemia**	Yes	7.0 ± 5.3	0.224
	No	10.6 ± 10.7	
**Mental health (depression, anxiety)**	Yes	13.95 ± 13.78	<0.001 ***
	No	10.36 ± 10.39	
**Hypertension and kidney disease**	Yes	10.62 ± 11.74	0.947
	No	10.55 ± 10.57	
**Chest pain**	Yes	13.2 ± 13.5	0.087
	No	10.5 ± 10.5	
**Diabetes**	Yes	8.2 ± 8.5	0.195
	No	10.6 ± 10.6	
**Lifestyle Factors**			
**Alcohol**	Yes	10.5 ± 10.6	0.403
	No	10.9 ± 10.7	
**Exercise**	Yes	10.5 ± 10.5	0.119
	No	11.2± 10.8	
**Smoking**	Yes—current smoker	12.2 ± 11.5	<0.001 ***
	Yes—ex-smoker	11.99 ± 10.6	
	No	8.9 ± 9.4	

* *p* < 0.05; *** *p* < 0.001.

**Table 4:** Negative binomial regression of the predictors of number of sick leave episodes.

	Incidence Rate (IR)	95% CI	*p*-Value
**Sex**—ref. Male Female	1.24	1.14, 1.36	<0.001 ***
**Age**—ref. <35 years ≥35 years old	1.19	1.08, 1.30	<0.001 ***
**Marital status**—ref. Not married Married	1.21	1.11, 1.33	<0.001 ***
**Grade**—ref. G9 to G13+			
G1 to G8	1.52	1.39, 1.67	<0.001 ***
**Medical History**—ref. No			
Mental health	1.24	1.03, 1.48	0.021 *
Gastrointestinal problems	1.23	1.13, 1.34	<0.001 ***
Respiratory problems and allergies	1.28	1.17, 1.40	<0.001 ***
Back pain	1.36	1.24, 1.49	<0.001 ***
Chest pain	1.02	0.78, 1.33	0.892
Diabetes	0.86	0.59, 1.26	0.441
Headache	1.23	1.07, 1.41	0.003 **
**Lifestyle factors**			
**Exercise**—ref. No exercise			
Exercise (yes)	1.03	0.95, 1.13	0.448
**Smoking**—ref. No smoking			
Smoking—current smoker	1.21	1.11, 1.32	<0.001 ***
Smoking—ex-smoker	1.12	0.83, 1.50	0.467

* *p* < 0.05; ** *p* < 0.01; *** *p* < 0.001.

## Data Availability

The data presented in this study are available on request from the corresponding author.
